# Chromatin Modifiers in Transcriptional Regulation: New Findings and Prospects

**DOI:** 10.32607/actanaturae.11101

**Published:** 2021

**Authors:** M. Yu. Mazina, N. E. Vorobyeva

**Affiliations:** Institute of Gene Biology RAS, Group of transcriptional complexes dynamics, Moscow, 119334 Russia

**Keywords:** transcription, chromatin, enhancer, co-regulator, remodelling, transcriptional factor

## Abstract

Histone-modifying and remodeling complexes are considered the main coregulators that affect transcription by changing the chromatin structure. Coordinated action by these complexes is essential for the transcriptional activation of any eukaryotic gene. In this review, we discuss current trends in the study of histone modifiers and chromatin remodelers, including the functional impact of transcriptional proteins/ complexes i.e., “pioneers”; remodeling and modification of non-histone proteins by transcriptional complexes; the supplementary functions of the non-catalytic subunits of remodelers, and the participation of histone modifiers in the “pause” of RNA polymerase II. The review also includes a scheme illustrating the mechanisms of recruitment of the main classes of remodelers and chromatin modifiers to various sites in the genome and their functional activities.

## INTRODUCTION

**Fig. 1:**
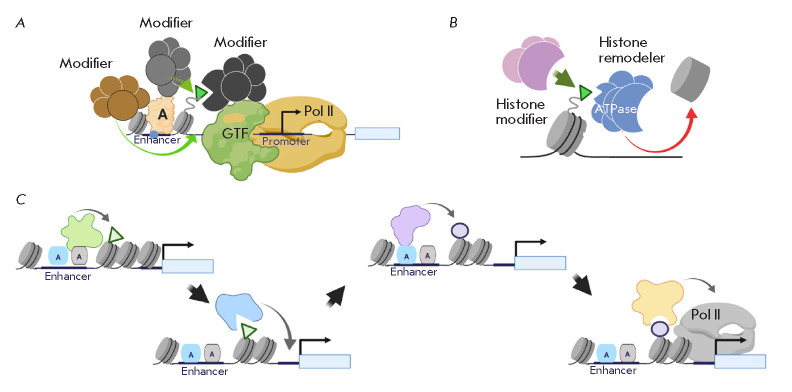
(*A*) – general model of transcriptional activation of eukaryotic genes. A –transcriptional activator; GTF –General transcriptional factors; Pol II –RNA polymerase II. (*B*) –the main classes of chromatin modifiers: chromatin remodeling complexes and covalent histone-modifying complexes. (*C*) –“Ratchet-clock” model of transcriptional regulation. According to the “Ratchet-clock” mechanism, covalent histones modifications mediate the change of transcriptional complexes at the regulatory regions (play the role of connecting elements in transcription regulation). A more detailed description of the figures is given in the text. There are also references to the works that served as the basis for the molecular models. All illustrations were created using the app BioRender.com

The general activation process of eukaryotic gene transcription begins with the binding of an activator protein (for example, a hormone receptor) to a regulatory element. The activator protein, with the help of protein complex coregulators, promotes the recruitment of general transcription factors (GTFs) to the gene. Multiprotein coregulatory complexes coordinate the transcription process; they integrate signals from various DNA-binding activators and chromatin modifications and transmit them to GTFs (*Fig. 1A*). The principal coregulatory complexes involved in the transcription of any gene are chromatin modifiers. They are divided into two large, functionally different groups: complexes that change the position of nucleosomes and those that covalently modify histones in chromatin (*Fig. 1B*).

It is known that hundreds of different proteins are involved in the activation of transcription. Apparently, they cannot bind the regulatory elements of the activated gene simultaneously throughout the entire process of transcription activation (although this possibility had been previously assumed as part of the “histone” code hypothesis). Today, it is customary to describe the transcriptional process as extremely dynamic. Moreover, different coregulatory complexes are thought to be responsible for each of its many stages. This model mechanism of transcription regulation is called the “ratchet-clock mechanism” (*Fig. 1C*) [1]. According to this model, the intermediate markers regulating the directed exchange of transcriptional complexes at the DNA regulatory elements are covalent histone modifications [2, 3]. Covalent modifications can promote not only the recruitment, but also the removal of transcriptional complexes from the regulatory element, thereby stimulating the dynamics of the transcriptional process. It has been shown that a decrease in the time of association of transcriptional regulators with DNA enhances transcriptional activation [4]. A positive feature of the “ratchet-clock mechanism” model consists in that it illustrates the possibility of a large number of proteins functioning on a single regulatory element of a gene. The preservation of information from previously recruited coregulators in the form of a modification on chromatin allows the organism to maintain the general direction of the regulated process (movement towards the active work of the regulatory element or, conversely, suppression of its activity).

**Fig. 2:**
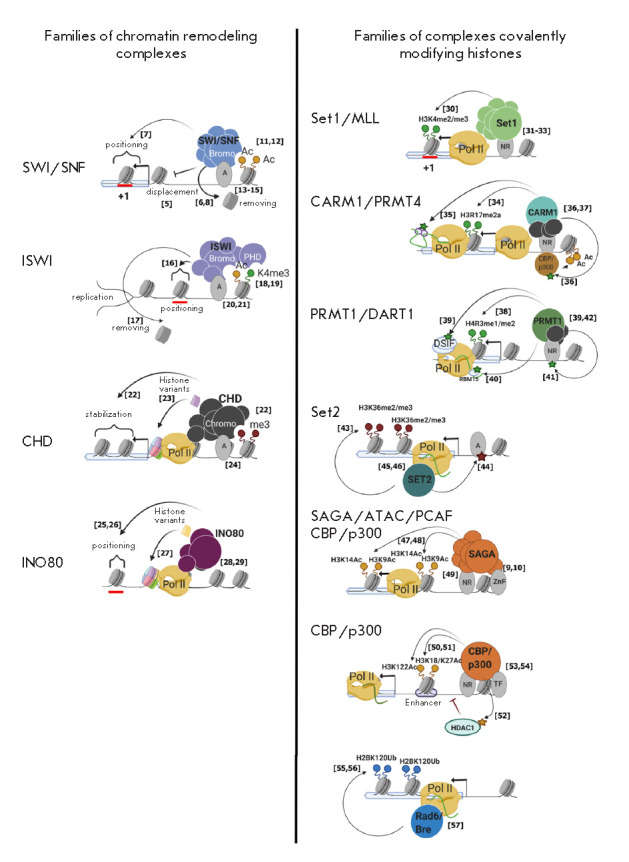
Functional features and mechanisms of recruitment for histone remodelers and histone modifiers into chromatin. Abbreviations: A – activator, NR – nuclear receptor, TF – transcription factor. All models were created using the app BioRender.com

This review aims to summarize the available information on the functional properties and recruitment mechanisms of chromatin modifier complexes (this information is summarized in the form of a diagram in *Fig. 2*, which includes references to scientific studies describing the individual properties of the chromatin modifiers). In more detail, we describe those areas of study pertaining to chromatin modifiers that have advanced significantly in recent years. In addition, we discuss a number of issues that have not yet been resolved.

## THE MOST ACTIVE FIELDS IN THE STUDY OF COACTIVATORS AFFECTING CHROMATIN

**Transcriptional complexes that change the positions of nucleosomes**

Since the emergence of chromatin (DNA packaged into fibrils using histone proteins) in the course of evolution, the most important way to regulate gene transcription has been to influence chromatin packaging, determining the availability of regulatory DNA elements. The protein complexes called chromatin remodelers belong to the transcriptional coregulators that affect the chromatin state [58, 59]. These transcriptional complexes are evolutionarily conserved (i.e., they are present in all eukaryotic organisms, from yeast to humans). Although the subunit composition of these complexes changes during evolution, their molecular properties (i.e., their ability to influence the position of nucleosomes in a certain direction) and the composition of their core subunits remain practically unchanged.

**The molecular mechanisms of pioneer factors**

DNA-binding transcription factors play the main role in the specificity of eukaryotic transcriptional regulation. It is the set of transcription factors associated with the regulatory element that affects its type of activity (which is realized by recruitment of various transcriptional complexes). It is a generally accepted fact that most transcription factors (for example, nuclear receptors) cannot bind to the regulatory DNA region occupied by nucleosomes. It is believed that a special class of DNA-binding proteins called pioneer factors is responsible for the displacement of compacted chromatin from regulatory DNA elements; the FoxA and GATA factors are prominent examples of this class [60]. These pioneer factors have a special property: the ability to bind regulatory DNA elements in a state of compacted chromatin and bring them into a state competent for binding by other transcription factors. Thus, pioneer factors are, in essence, the primary regulator-remodelers, initiating changes in the chromatin structure, which is further supported by transcriptional remodeling complexes. Despite the fact that the concept of pioneer factors was formulated almost 10 years ago, the molecular mechanism of the functioning of these proteins remains not fully understood. Initially, it was thought that pioneer factors function on their own, without the participation of remodeling transcriptional complexes (this assumption was based on the ability of these proteins to bind chromatinized DNA *in vitro*) [61]. At the same time, it has long been noted that pioneering factors *in vivo *are capable of affecting chromatin in quite complex ways (for example, replacing histones H2A with H2AZ), which is hardly possible for individual monomer proteins [62].

**Fig. 3:**
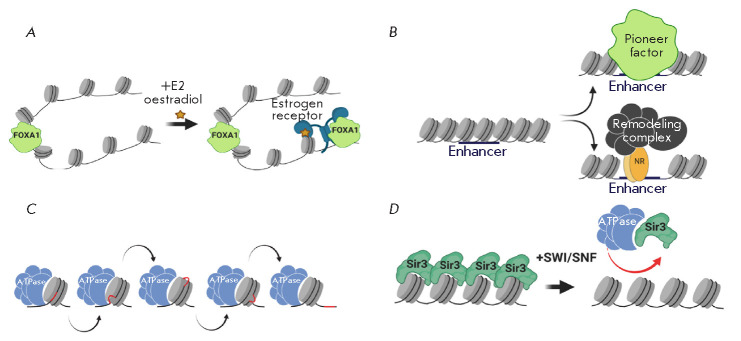
(*A*) – cooperative work of the pioneer factor FoxA1 and the nuclear receptor ERα during the chromatin de-compaction at the DNA regulatory sites. (*B*) – the primary binding of transcriptional regulators to chromatinized DNA elements can be carried out both by specialized DNA-binding factors-“pioneers” or the chromatin remodeling complexes associated with the nuclear receptors. (*C*) –all families of remodeling complexes operate via the same molecular mechanism: the formation of a DNA loop on the nucleosome and the change of its position relative to the nucleosome surface. (*D*) –effect of SWI/SNF chromatin remodeler on the binding of the Sir3p repressor to chromatin. The SWI/SNF complex in yeast is able to interact with the heterochromatin repressor Sir3p and remove it from chromatin. A more detailed description of the figures is given in the text. There are also references to the works that served as the basis for the molecular models. All illustrations were created using the app BioRender.com

According to current views, it is unlikely that pioneer factors function as single proteins in living cells. Most likely, their unique ability to act on compacted chromatin is a consequence of cooperative multiprotein interactions. An example of such joint functioning can be the paired work of a pioneer factor with a nuclear receptor (for example, the pioneer factor FoxA1 and the nuclear receptor ERα) [63]. It has long been known that the binding of FoxA1 and ERα to DNA occurs cooperatively. However, it was assumed that the pioneering factor plays a leading role in this process, as it is the suppression of FoxA1 expression that leads to the removal of 90% of ERα genomic sites with a very weak reverse effect in a reciprocal experiment [64]. Nevertheless, further studies have shown a more significant role for nuclear receptors in chromatin de-compaction at DNA regulatory sites. In fact, oestradiol treatment (of which ERα is a sensor) of MCF-7 cells leads to an increase in the number of FoxA1 binding sites by almost 30%, thereby demonstrating the ability of ERα to act as a pioneer factor, at least for some FoxA1 sites (*Fig. 3A*) [65]. Most likely, the ability of a nuclear receptor to play the role of a pioneer factor may be based on its ability to interact with transcriptional complexes and chromatin remodelers. It is known that many steroid receptors use SWI/SNF and NURF remodeling complexes to de-compact chromatin at the early stages of gene transcription activation [66, 67]. There is a hypothesis about the possibility of the formation of a common complex between the nuclear receptor and the transcriptional remodeler complex not on chromatin but in nucleosol [67]. Such a pair would be an effective pioneer factor capable of interacting with the regulatory regions within compacted chromatin (*Fig. 3B*). Further research is needed to understand how common this molecular mechanism is in nature.

**The functional activity of chromatin remodeling complexes. The possibility of remodeling non-histone proteins**

While the mechanism that organizes primary access to the regulatory elements of compacted chromatin remains unclear, the maintenance of nucleosome-free regions is undoubtedly the responsibility of chromatin remodeling complexes. In general, transcriptional remodeling complexes can affect nucleosomes in a variety of ways: to remove them, shift, position, or replace histones with alternative variants. However, all these mechanical functions are based on the same ability of remodelers to create a DNA loop within the nucleosome and change its position relative to the nucleosome’s surface (*Fig. 3C*) [58]. The subunit composition of the remodeling complexes, as well as the structural features of the ATPase subunits (the presence of additional domains that are capable of binding a certain type of histone), determines the functional ability of the corresponding transcriptional complexes. Thus, complexes of the SWI/SNF family, which carry the SnAC domain that binds nucleosomes in their enzymatic subunit, are responsible for the removal of entire nucleosomes from chromatin [68]. Complexes of the INO80 family, which have a two-part translocation domain in their ATPase, are capable of replacing histones in nucleosomes with alternative variants [27]. The ISWI ATPase family, having a C-terminal HSS domain that binds unmodified histone H3 and regions of linker DNA, participates in the coreplicative assembly of chromatin, helping chaperones form high-grade nucleosomes within chromatin [69]. In addition, remodeling complexes of the ISWI and CHD families use their HSS and DBD domains for accurate postreplicative positioning of nucleosomes in chromatin [16].

It should be noted that chromatin remodeling complexes can directly influence not only the position of nucleosomes on DNA, but also the association of other DNA-binding proteins with chromatin [70]. The ability of remodeler translocation domains to bind and induce the movement of transcription factors and transcription repressors may play a significant role in the regulation of gene transcription. Thus, it was found that the ATPase of the SWI/SNF complex in yeast is capable of interacting with the heterochromatin repressor Sir3p and removing it from nucleosome templates *in vitro* [71]. More recently, it was proven *in vivo *that the SWI/ SNF complex participates in the removal of the repressive effect of Sir3p from its target genes during the activation of their expression in the M/G1 phase of the cell cycle (*Fig. 3D*) [72].

**Fig. 4:**
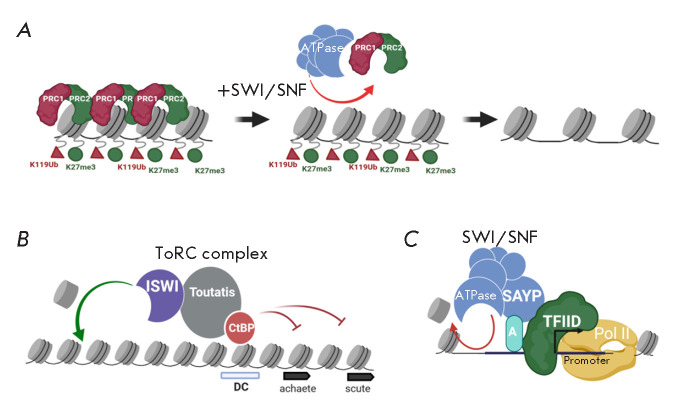
(*A*) – artificial SWI/SNF recruitment leads to the decrease in PRC binding at the repressed loci. (*B*) – the ISWI remodeler, as part of the ToRC repressor complex of Drosophila, interacts with the transcriptional repressor CtBP. CtBP enhances the remodeling properties of ISWI, and ISWI is involved in the repression of the transcription of CtBP-dependent genes. (*C*) – the SAYP subunit of SWI/SNF mediates the recruitment of both SWI/SNF and TFIID to the genomic sites. A more detailed description of the figures is given in the text. There are also references to the works that served as the basis for the molecular models. All illustrations were created using the app BioRender.com

The functional role of the SWI/SNF remodeling complex in the withdrawal of Pc-driven repression has been demonstrated as relates to various organisms [73]. The positive correlation in the violation of these molecular systems during oncotransformation of cells has been studied extensively [74]. Until recently, it was believed that the role of SWI/SNF complexes in the removal of PRC complexes from chromatin may be indirect. However, recent experiments on the artificial recruitment of SWI/SNF to the Pc-repressed locus have demonstrated direct removal of PRC complexes by the SWI/SNF complex (artificial recruitment of the latter led to a decrease in the PRC level within several minutes and did not depend on the recruitment of RNA polymerase II to the studied locus) (*Fig. 4A*) [75]. The role of remodeling complexes in the removal of transcription factors from chromatin is likely much more significant than is currently known. Unfortunately, the study of this mechanism *in vivo *is an extremely complicated methodological problem. The obtained information can almost always be questioned because of the presence of indirect experimental contributors.

**The noncatalytic function of remodeling complexes in the regulation of transcription**

Many transcriptional chromatin remodeling complexes are characterized by the presence of a large number of subunits, in addition to the enzymatic subunit responsible for histone movement [76]. Moreover, the number of subunits in these complexes increases over the course of evolution [77]. Previously, it was believed that the noncatalytic subunits of chromatin remodeling complexes are responsible for the specificity of recruitment to chromatin. It has been shown that a decrease in the intracellular level of individual noncatalytic SWI/SNF subunits of Drosophila leads to a complete disruption of the binding of this complex to chromatin, while preserving the structural stability of its core module that contains ATPase [78]. Recently, the attitude of researchers towards the functional capabilities of the noncatalytic subunits of remodelers has changed. There are data indicating the presence of additional functions in the noncatalytic subunits of chromatin remodeling complexes.

This development appears quite logical from an evolutionary point of view. Transcriptional activation and repression are extremely dynamic and complex processes. Within these processes, many multicomponent complexes replace each other at high speed in a limited space (i.e., on regulatory DNA elements). This exchange assumes a high probability of contacts between the participants and, accordingly, the possibility of positive or negative mutual regulation. Chromatin remodeling complexes, in the course of their work on a regulatory element, bring with them many additional noncatalytic subunits. It is likely that while the ATPase part of the complex performs its main catalytic activity, the remaining subunits participate in the activation/ repression of the transcription process [79]. The best characterized is the association of the ATPase subunits of remodeling complexes with transcriptional repressors. During the study of the ToRC repressor complex of Drosophila, the ability of the ISWI enzymatic remodeler subunit to physically interact with the transcriptional repressor CtBP was described [80]. Moreover, the ATPase and repressor subunits in this complex were discovered to exert a reciprocal functional effect on each other: CtBP enhances the ability of ISWI to remove or insert nucleosomes, and ISWI is apparently involved in the transcriptional repression of CtBP-dependent genes (*Fig. 4B*). Another remodeler enzyme, the chromodomain-containing ATPase CHD4/ Mi-2, was also shown to be able to interact with other proteins to form the NuRD complex, which represses gene transcription [81]. This repressor complex contains more subunits than the ToRC complex described above. NuRD subunits form dynamically interacting modules with the remodeling activity implemented by the CHD4/Mi-2 subunit or the histone deacetylase activity due to Rpd3 [82]. The functional role of NuRD includes controlling for both the density of nucleosomes and their level of covalent modifications at developmental enhancers [83].

Interestingly, the ATPase subunit of the SWI/SNF complex, the BRM protein, was also found to exert a repressive effect on transcription, independent of its catalytic activity [84, 85]. At the moment, it is unclear which molecular partners enable the repressive functions of BRM ATPase. However, it seems reasonable to propose a mechanism for the positive role of SWI/ SNF in transcription regulation that does not depend on the ATPase activity of this complex. Approximately ten years ago, the physical interaction of the Drosophila SWI/SNF complex with the common transcription factor TFIID, mediated by its SAYP subunit, was described [79, 86]. It was shown that the SAYP subunit plays a key role in the recruitment of the SWI/SNF complex to half of its genomic targets [87]. Interaction with TAF5 allows SAYP to recruit not only the SWI/ SNF remodeling complex, but also TFIID to its genomic targets, contributing to the formation of the preinitiation complex (*Fig. 4C*) [79, 88, 89]. Thus, the noncatalytic SWI/SNF subunit is a bifunctional regulator that simultaneously promotes chromatin remodeling and transcription initiation.

**The transcriptional complexes that covalently modify histones**

Since the inception of the “histone code” hypothesis, proteins capable of covalent modification of histones have been the subject of numerous studies [90]. For a long time, it was assumed that the set of histone modifications determines the pattern of transcriptional complexes associated with the regulatory elements of the genome (which is the concept of the “histone code”). Currently, researchers are inclined to believe that the presence of certain chromatin modifications is a sufficient condition for the recruitment of only a limited number of regulators [1]. In most cases, the binding of histone modification is only an additional factor in the recruitment of the transcriptional regulator or may not even contribute at all to its recruitment to chromatin.

**The role of covalent histone modifications in the recruitment of transcriptional complexes to chromatin**

Initially, the “histone code” hypothesis was actively investigated in the context of the transcription activation process. Many researchers tried to establish the histone modifications that determine the recruitment of the protein complexes stimulating transcription. In turn, researchers who studied protein complexes worked to determine the protein domains responsible for the recruitment of the complexes to the corresponding “activating” modification. It is worth noting that many of these studies proved unsuccessful. It turned out that such “activating” histone modifications are often unable to recruit transcription complexes by themselves. A striking example of such an “activating” modification with a complex history of its study is the trimethylation of histone H3 at position 4. Indeed, there is much evidence of the correlation between the presence of this modification on the promoter and the active work of the corresponding gene [91]. However, the role of this modification in the recruitment of transcriptional regulators to the promoters of the corresponding genes is not so unambiguous. Domains capable of specifically interacting with the H3K4me3 modification have been identified in various protein complexes (among which the TFIID, NURF, mSin3a –HDAC1, and SAGA complexes are especially noteworthy) [92, 93]. For the first time, a specific domain that binds the H3K4me3 modification was identified in the ING2 protein, which is part of the mSin3a –HDAC1 repressive complex [94]. However, it was shown almost immediately that the disruption of the interaction between ING2 and the modification of histone H3K4me3 leads to a change in the functional activity of the complex (a decrease in deacetylating activity) rather than to a violation of its recruitment [95]. The study of the domain that recognizes the H3K4me3 modification in the CHD1 chromatin regulator developed in a similar fashion [96]. The specific interaction of CHD1 with this chromatin modification disrupts the functional activity of the complex but does not prevent its interaction with chromatin [97]. It should be noted that, in the case of the TFIID and NURF coregulators, a positive contribution of the protein domains recognizing the H3K4me3 modification to the recruitment of these complexes to genomic sites was demonstrated [98, 99, 100].

**Fig. 5:**
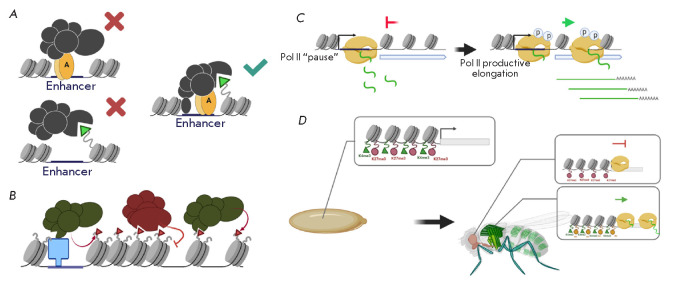
(*A*) – the combinatorial nature of the recruitment of transcriptional co-regulators. The coregulator subunits often include DNA-binding motifs, domains recognizing covalent histone modifications, as well as domains of association with nuclear receptors and transcription factors. A number of protein domains can play a role in the association of a coregulator with a regulatory DNA element, as well as affect its functional activity. (*B*) – general concept of the role of histone modifications in the propagation of compacted chromatin. The initial recruitment of chromatin-compacting complexes is mediated by the DNA-binding factors. Covalent histone modifications are involved in a process of propagation of chromatin compaction around the site of the initial binding. (*C*) – The “pause” state of RNA polymerase II is characterized by the presence of short “abortive” transcripts at the promoter-proximal regions of inactive genes. (*D*) –genes with bivalent histone modifications (both, active, H3K4me3, and repressive, H3K27me3) in embryogenesis have enhanced capability of duality of action later in development (to be repressed or activated depending on the type of tissue they present). A more detailed description of the figures is given in the text. There are also references to the works that served as the basis for the molecular models. All illustrations were created using the app BioRender.com

Apparently, the process of recruitment of protein complexes to the regulatory elements of DNA is more complicated than we had imagined earlier: it is not realized through individual protein-protein interactions (for example, between a histone modification and a separate protein domain that “reads” the modification or between a DNA-binding transcription factor and a subunit of the protein complex). The protein complexes that regulate transcription most often contain a set of different subunits, many of which carry different protein domains (i.e., domains that are capable of DNA binding, recognize histone modifications, and interact with transcription factors). It appears that several domains that are part of various subunits are involved in a single act of recruitment of a transcriptional complex to chromatin. It is the set of such DNA-protein and protein-protein interactions that are realized in a separate act of recruitment of a complex to a regulatory element that can determine the type of functional activity of the complex in a given chromatin region (*Fig. 5A*).

The role of covalent histone modifications in the spreading of compacted chromatin, which represses transcription, has been investigated much more in detail and unambiguously. The cell uses various systems to create regions of compacted chromatin to suppress unwanted gene transcription. Two active systems of chromatin compaction can be distinguished based on the Pc and HP1 proteins, both containing chromoprotein domains capable of binding specific methylated residues of histone H3 [101, 102, 103]. Interestingly, for both chromatin compaction systems (Pc- and HP1-dependent), recognition of covalent histone modifications plays a role precisely at the stage of chromatin spreading within the chromosomal domain but not at the stage of primary recruitment of repressive complexes to DNA (which is carried out by specific DNA-binding proteins) (*Fig. 5B*). Thus, the propagation of Pc-dependent repression occurs with the participation of the PRC1 and PRC2 complexes, one of which is capable of recognizing the H3K27me3 chromatin modification, while the second introduces it. The interrelated molecular work of these complexes organizes the propagation of Pc-dependent repression around the PRE elements, which are initiators of Pc-dependent compaction [104]. Apparently, the histone modification of H3K27me3 is necessary for not only the propagation of Pc-dependent chromatin along the DNA strand, but also for the preservation of the corresponding chromatin status after the replication fork has passed through it [105]. A positive feedback loop based on the introduction of a covalent histone modification also exists in the mechanism of pericentromeric heterochromatin spreading. In this case, methyltransferase Su(var)3-9 (Suv39H in mammals) modifies histone H3 at position 9, which leads to the recruitment of the HP1 heterochromatin protein (which in turn recruits a new portion of methyltransferase to the compacted site) [106].

In the processes of activation and repression of transcription, the recognition of histone modifications is often not the primary signal that determines the recruitment of transcriptional regulators. The logical extension of the “histone” code idea was the hypothesis holding that covalent modifications of histones are necessary for the exchange of transcription complexes at regulatory sites [107]. This was facilitated by experiments that showed the existence of an active exchange of nucleosomes and associated proteins on working regulatory elements [108].

**The role of covalent histone modifications in the regulation of the RNA polymerase II pause**

For a long time, the recruitment of RNA polymerase II to promoters was considered the main mechanism of activation of gene transcription. Later, it became obvious that many inactive genes of multicellular organisms contain bound RNA polymerase II on their promoters [109]. The transcription of such genes is activated by stimulating the productive elongation of RNA polymerase II transcription. This mechanism of transcriptional regulation is called the “pause” of RNA polymerase II and is characterized by the presence of short “abortive” transcripts on the promoters of inactive genes (*Fig. 5C*). Currently, it is believed that this mechanism is widely used by organisms to regulate the transcription of genes that require high accuracy of induction in space and time (for example, in a certain tissue or developmental stage) [110]. The prevalence of this mechanism has made it an attractive area of research. One of the intensive areas of research on RNA polymerase II pausing was the search for the covalent histone markers associated with both the “pause” itself and the release of RNA polymerase II from this state.

For instance, the first description of bivalent nucleosomes was provided in the context of studying the “pause” of RNA polymerase II [111]. In mouse embryonic stem cells, it was found that the RNA polymerase II “pause” is present on promoters carrying the H3K27me3 modification in chromatin, which is characteristic of transcriptional repression. At the same time, RNA polymerase II was absent on promoters carrying both active H3K4me3 and repressive H3K27me3 modifications (containing bivalent nucleosomes) [112]. Later, it became clear that bivalent modifications in embryonic stem cells are mainly present on the promoters of genes whose transcription is regulated in different ways during cellular differentiation [113]. During development, these genes are activated in certain tissues (an active modification of H3K27Ac is introduced to their promoters), while in others, they remain inactive (the H3K4me3 modification is removed from their promoters, H3K27me3 is preserved, and the genes are put into a state of transcriptional “pause”) (*Fig. 5D*) [114]. This concept has been supported by various data. The maintenance of the Pol II “paused” state and the transfer of promoters to this state were found to be carried out by enzymes that modify the K4 and K27 residues of histone H3. Thus, the maintenance of the “pause” state on gene promoters in mouse embryonic stem cells was associated with the activity of Lsd1-specific demethylase H3K4me3 [115]. For the JMJD3 enzyme, which is aimed at demethylation of the H3K27me3 modification, a role in the control of transcription elongation in human cells was also revealed [116]. It was shown that a decrease in the intracellular level of this demethylase leads to a decrease in the level of elongating RNA polymerase II.

There are a number of covalent histone modifications that are associated with the release of RNA polymerase II from a “pause” state and the stimulation of transcription elongation. The acetyl residues of histones are positive markers of transcription elongation. This role (to overcome the transcription pause and stimulate elongation) was discovered for the main acetyltransferase, functioning on enhancers, the CBP protein [117]. CBP was found to introduce acetylation at H3K27 of the first nucleosome in the gene's body, which is essential for the elongation of RNA polymerase II. Another acetyl modification of histones, H3K9Ac, was associated with the release of RNA polymerase II from the “pause” state by recruitment of the SEC (super elongation complex), which contains a number of the factors necessary for transcription elongation [118]. A decrease in the level of H3K9Ac was shown to prevent the elongation of genes and to lead to an increase in the “pause” index (i.e., an increase in the ratio between the levels of RNA polymerase II on the promoter and in the gene's body).

**Fig. 6:**
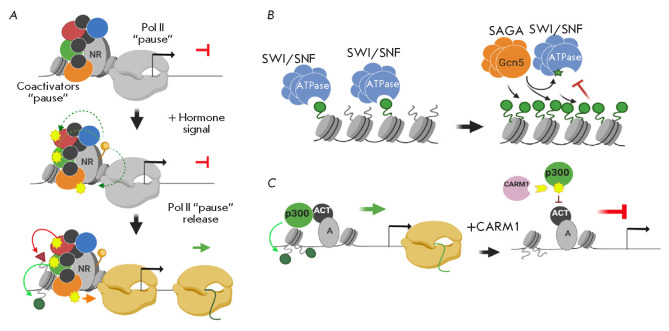
(*A*) – promoter regions of genes regulated via Pol II pausing contain not only pre-bound Pol II, but also prebound co-activators in their inactive state. Transcriptional induction is realized with the transition of co-regulators into the functional state but not with an increase in their promoter-bound level. (*B*) – the SAGA histone acetyltransferase complex acetylates the ATPase subunit of the SWI/SNF complex, regulating its ability to bind chromatin. (*C*) – arginine methyltransferase CARM1 methylates the CBP/p300 acetyltransferase, decreasing the activity of CBP/p300 and disrupting its ability to bind transcription activators. A more detailed description of the figures is given in the text. There are also references to the works that served as the basis for the molecular models. All illustrations were created using the app BioRender.com

Recently, our group has studied the kinetics of recruitment of chromatin modifiers and the appearance of covalent histone modifications in the first minutes of transcriptional activation (on a model of developmental genes, which persist in a “pause” state in *Drosophila* cells) [119]. We have studied the recruitment of two dozen transcriptional complexes, which allowed us to identify an unexpected regulatory effect. We almost did not observe an increase in the level of binding of chromatin-modifying complexes with promoters during their activation. At the same time, we found a significant increase in the level of chromatin modifications introduced by these complexes. We called this effect the “pause” of transcriptional coactivators (*Fig. 6A*). We believe that during the formation of a transcriptional “pause,” not only RNA polymerase II, but also many coregulatory complexes that modify chromatin are recruited to the promoters. The signal-inducing transcription does not lead to a further increase in the level of binding of these complexes but stimulates their functional activity, leading to an increase in the level of chromatin modifications. We plan to test the prevalence of the effect of coactivator “pause” in the* Drosophila *genome in future studies.

**Are covalent histone modifications actually “side targets”?**

The covalent modifications of histones have long attracted the attention of researchers because of the popularity of the “histone code” hypothesis. In particular, the introduction of covalent histone modifications was described as the main molecular function for a variety of transcriptional regulators (including multisubunit complexes). Later, it was discovered that a number of histone modifications make rather modest functional contributions to the regulation of transcription and that the enzymatic functions of the regulators introducing them have completely different, nonhistone protein targets, which are of greater importance.

A striking example of such a chromatin modifier is the SAGA complex, which is capable of acetylating lysine residues in the histones H3 and H4. For a long time, researchers believed that the acetyl groups introduced by this complex are specific labels that are accurately “read” by other transcriptional regulators using protein “reader” domains. In particular, it was assumed that the modifications introduced by the SAGA complex are recognized by bromodomains, which are part of the SWI/SNF complex, which acquires the ability to remodel exactly acetylated histones [120]. This hypothesis was in good agreement with the joint presence of SAGA and SWI/SNF complexes at the genomic sites of various organisms [10, 121]. Over time, it became clear that the acetyl residues of histones are unlikely to be specific markers for the recruitment of any specific complexes. The point is that the functional effect of acetyl chromatin residues on transcription has a combinatorial nature that depends on the total number of modified residues but is almost indifferent to their qualitative composition [122, 123]. Deeper studies have led to the description of additional targets for acetylation by the SAGA complex. In particular, SAGA acetylates the ATPase subunit of the SWI/SNF complex, thereby regulating the strength of its binding to chromatin (*Fig. 6B*) [124].

Additional protein targets have been described for other chromatin modifiers. Thus, the arginine methyltransferase CARM1, originally described as a specific modifier of 17-arginine in histone H3, was found to methylate the arginine residues of many transcriptional regulators, modulating their functions [125, 126]. In particular, the targets of CARM1 activity are splicing factors, and methylation provokes exon skipping in mRNA [35]. Another target of CARM1 methylation is CBP/p300 acetyltransferase, which is one of the key enzymes that function on enhancers. Methylation of CBP/p300 by CARM1 decreases acetyltransferase activity and impairs its ability to bind transcriptional activators (*Fig. 6C*) [36, 127].

As we can see, a deeper study of transcriptional regulators, initially characterized as chromatin modifiers, leads to the description of their additional enzymatic targets. It is likely that further study of these additional targets will uncover a higher functional significance in comparison with target histones, which for a number of modifiers can only be “side targets.” This assumption is supported by the results of some mutational studies that aimed at identifying the functional significance of individual histone modifications. It has been shown that mutations in individual chromatin modifiers have a stronger effect on the regulation of transcription than mutations in their target sites in histones, thereby demonstrating the presence of more significant targets –transcriptional regulators [128, 129]. It is likely that future studies will reveal other histone modifications that are only a by-product of the action of the chromatin modifier, achieving its main regulatory target.

## FURTHER PROSPECTS AND UNRESOLVED ISSUES

The growing volume of experimental information on the mechanisms of transcriptional regulation and the activity of coregulators has not led to answers to some of the questions that were formulated earlier. Below, we will address several problems for which definite solutions have yet to be found, despite the wealth of experimental arsenals available today.

**Recruiting a transcriptional regulator to chromatin: in a complex with a DNAbinding protein or sequentially?**

**Fig. 7:**
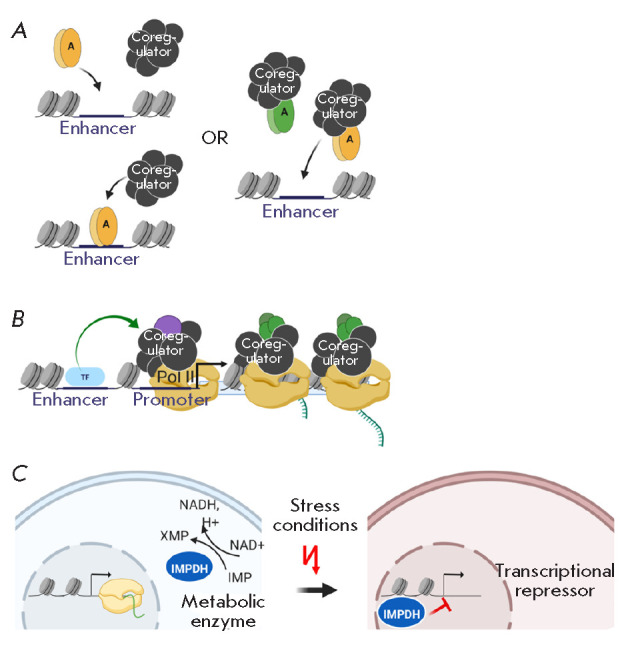
(*A*) – recruitment of a transcriptional regulator to chromatin: in a combination with a DNA-binding protein or stepwise. (*B*) – changes in the subunit composition of protein complexes during the transcriptional cycle: transformation of the same complex or recruitment of a new complex. (*C*) – inosine-5’-monophosphate dehydrogenase (IMPDH), an enzyme of purine biosynthesis that works in the cytoplasm of the cell, is able to shuttle into the nucleus under stress conditions and regulate gene transcription. A more detailed description of the figures is given in the text. There are also references to the works that served as the basis for the molecular models. All illustrations were created using the app BioRender.com

DNA-binding proteins determine the specificity of the effect of the coregulator on gene transcription. They mediate the binding of coregulators to enhancer and promoter sequences. Until now, the general mechanism of interaction of coregulators with DNA has remained unclear. Does the sequential binding of the DNA-binding protein to the regulatory element and the subsequent recruitment of the coregulatory complex occur, or is the preformed complex recruited to the genomic sites? (*Fig. 7A*).

The concept of sequential recruitment looks questionable in the context of studies focused on the dynamics of protein binding to chromatin. It was shown that the association of any proteins with DNA only lasts a few minutes [107]. In this regard, sequential association of proteins on the regulatory element looks unlikely – there remains very little time for their functional action. The hypothesis of the simultaneous recruitment of coregulators and DNA-binding proteins has been invoked repeatedly for a long time. Nevertheless, the initial concept of sequential binding appears more widespread [67]. However, it has been shown recently that complexes of transcription factors — nuclear receptors with chromatin remodeling coregulators — are capable of interacting with chromatin, acting as “pioneer” factors [130]. Moreover, it was demonstrated that knockouts of the ATPase subunits of the SWI/ SNF and ISWI coregulators significantly disrupt the binding of transcription factors in the genome of mouse embryonic stem cells (which would be impossible in the concept of sequential recruitment) [131]. All these data substantiate the model of joint recruitment of coregulators and DNA-binding factors. Biochemical isolation of transcription factors, in combination with coregulators, would be very useful to further lend credence to this concept. However, the connection between the transcription factor and the coregulator is a specific interaction, albeit a weak one, that is easily lost during biochemical purification. Let us hope that the recently developed techniques for studying weak protein-protein interactions (e.g., *in vivo *biotinylation of proteins by their partners) will help answer questions regarding the mechanisms underlying the interaction between coregulators and chromatin.

**Changes in the subunit composition of protein complexes during transcription: transformation of the same complex or recruitment of a new complex?**

Many coregulatory complexes are involved in the various stages of gene transcription. Often, in the course of the study of such complexes, researchers focus on examining the distribution and properties of the enzymatic subunits of the complex, while the behavior of the other subunits remains unexplored. Nevertheless, for a number of transcriptional complexes, it was shown that their composition is not constant and can change depending on the stage of gene transcription (*Fig. 7B*). Thus, it is known that the transcriptional coregulator SAGA exhibits acetyltransferase and deubiquitinylating activities towards histones. Both of these activities are required for SAGA to function on the gene promoter, where it promotes the initiation of transcription [132, 133]. At the same time, it is known that a component of the deubiquitinylating module of the SAGA complex, the SGF11 protein, is also associated with the CAP of newly synthesized mRNA as a part of the AMEX complex, where it is involved in mRNA export from the nucleus to the cytoplasm [134]. An interesting detail is the possibility of transition of the SAGA subunit SGF11 to the AMEX complex during transcription. Is there an independent recruitment of two separately existing complexes to the active gene? Or is there a subunit transformation of the SAGA complex, initially recruited to the promoter, during the transition of the RNA polymerase II complex into the body of the gene?

Another well-known example of a change in the subunit composition of a coregulator during transcription is the Mediator complex. The main role of this large multisubunit complex is to coordinate the recruitment of RNA polymerase to the promoter and initiate transcription [135]. However, the Mediator contains a separate four-subunit CDK8 module that possesses kinase activity and a number of additional functions. Interestingly, the interaction of the core Mediator with RNA polymerase II mutually excludes the presence of the CDK8 module. Moreover, the role of the CDK8 module in the stimulation of elongation, that is, in the latest stages of transcriptional activation, is well known [136]. It remains unclear how the module is recruited to CDK8-dependent genes in order to participate in elongation stimulation. Is this an alternative to Mediator- dependent recruitment, or is there a structural transformation of the entire Mediator complex during the transcriptional cycle?

The two examples given above are only an illustration of the challenges in the study of multisubunit complexes. There are many indirect confirmations of changes in the composition and properties of coregulatory complexes during transcription. However, there is still no direct experimental evidence of these phenomena due to a lack of convenient research methods.

**Influence of nontranscriptional complexes on transcription: hierarchy of functions and determination of the leading function**

The original strategy researchers employed to study the functions of proteins and protein complexes was to perform an in-depth study of one original function described for a protein of interest. Subsequently, another direction in the study of protein properties became more prominent, in which researchers tried to indentify and describe as many new functions as possible for a single protein, even when their molecular processes were sufficiently distant from each other. Therefore, a number of metabolic enzymes normally functioning in the cytoplasm of the cell were observed to shuttle into the cell nucleus under stress conditions and control gene transcription, acting as transcriptional regulators (*Fig. 7C*) [137]. Another impressive example is the ORC complex, which is responsible for recognizing the origins of replication and initiating the formation of a pre-replicative complex on DNA [138]. The ORC complex was recently shown to be involved in mRNA processing and transport from the nucleus to the cytoplasm. Many ORC subunits were shown to interact *in vivo *with processing factors, while their knockdown led to impaired mRNA transport [139, 140].

At some point, the problem of a rethinking of the available data and established views on the leading functions of some multifunctional complexes arises. It may well turn out that the initially described functional role for many regulators can only be an indirect result of their leading function, which was noticed much later. Given the exponential growth in the amount of experimental data, it is likely that we will have to go through such stages of rethinking of the hierarchy of functions for most of the known proteins. It seems to us that evolutionary research can be very helpful in this case. Obtaining information about the functional properties of proteins in non-model organisms can help trace the history of the emergence of new functions and create a hierarchy of their significance.

## CONCLUSION

Eukaryotic organisms use coregulatory complexes as one of the ways to control the transcription of a certain set of genes. Thus, coregulatory transcriptional complexes may well be promising therapeutic targets for the development of drugs aimed at altering the transcription levels of a specific set of genes. Currently, there are a number of such drugs in clinical trials. The following are considered the most promising transcription coregulator targets for the development of low-molecular-weight inhibitors: the EZH2 enzymatic subunit of the PRC2 complex, the Brd4 transcription elongation coregulator, and various HDAC histone deacetylases [141, 142, 143]. The development and testing of drugs aimed at modifying the functional properties of these proteins began quite recently. Of course, the family of transcriptional regulators still harbors many other promising target proteins.
